# Radiographic analysis and virtual cleaning of a bioarchaeological remain enclosed in mineral deposits from a limestone cave

**DOI:** 10.1186/s41747-020-00166-1

**Published:** 2020-07-09

**Authors:** Patrick E. Eppenberger, Mislav Čavka, Siniša Radović, Dalibor Paar, Nenad Buzjak, James C. M. Ahern, Philipp Biedermann, Philipp Gruber, Mario Novak, Ivor Janković

**Affiliations:** 1 Institute of Evolutionary Medicine, University of Zurich, Winterthurerstr, 190, CH-8057 Zurich, Switzerland; 2 Department of Diagnostic and Interventional Radiology, University Hospital Centre “Zagreb”, Zagreb, Croatia; 3 School of Medicine, Chair of Social Medicine and Organization of Healthcare, University Zagreb, Zagreb, Croatia; 4 Institute for Quaternary Paleontology and Geology, Croatian Academy of Sciences and Arts, Zagreb, Croatia; 5 Department of Physics, Faculty of Science, University Zagreb, Zagreb, Croatia; 6 Department of Geography, Faculty of Science, University Zagreb, Zagreb, Croatia; 7 Department of Anthropology, University of Wyoming, Laramie, WY USA; 8 Department of Neuroradiology, Kantonsspital Aarau, Aarau, Switzerland; 9 Institute for Anthropological Research, Centre for Applied Bioanthropology, Zagreb, Croatia

**Keywords:** Body remains, Croatia, Palaeontology, Radiometric dating, *Sus scrofa*, *T*omography (x-ray computed)

## Abstract

In limestone caves, environmental processes often cause alterations of human or animal skeletal remains, complicating classical analytical methods. Exemplary, a proximal femoral skeletal fragment, enclosed by a thick layer of speleothemic calcite deposits, was discovered during the exploration of the Bedara cave in Žumberak, Croatia. An examination without removal of the surrounding mineral deposits, possibly leading to damage of the specimen, was, therefore, desirable.

We describe and discuss the applied techniques, including clinical computed tomography, virtual cleaning by a specially developed segmentation protocol using an open-source DICOM viewer, and virtual visualisation and dimensioning using computer-aided design software, so that this “hidden” specimen could be non-invasively examined in great detail. We also report on the circumstances and origin of the find, the results of radiocarbon dating, and its anatomical and taxonomic identification, according to which, the bone fragment belonged to a wild boar (*Sus scrofa*) from the timeframe of the Middle Eneolithic Retz-Gajary culture in the region (4,781 ± 35 years before present). This study provides a reference for future paleontological and anthropological analyses, seeking to unlock the enormous potential of anatomical studies of comparable skeletal remains that are either petrified or enclosed in speleothemic deposits.

## Key points

- A novel virtual visualisation approach for fossilised skeletal remains non-invasively reveals their original shape and surface in great detail.
- We present a combination of computed tomography, a specially developed segmentation protocol, and virtual visualisation and dimensioning.
- Non-invasive methods of analysis leave bioarchaeological finds preserved for future generations.
- We provide a reference for future palaeontological and anthropological analyses, seeking to unlock the enormous scientific potential of fossilised skeletal remains.

## Background

### The Bedara cave and find circumstances

**Fig. 1:**
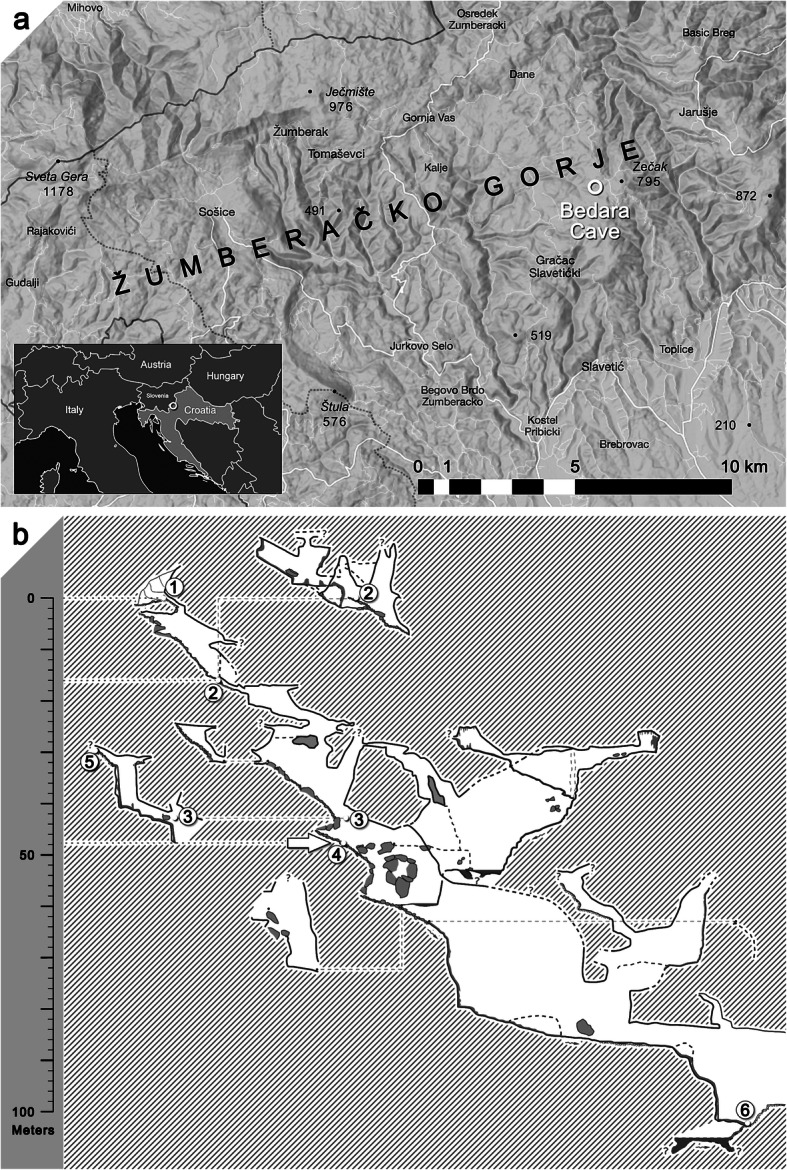
**a** Map of the Žumberačko Gorje region in northwestern Croatia. The Bedara cave is located on Mt. Žumberačka Gora. The area lies at the transition between the southeastern Alps, the northwestern Dinarides and the Pannonian Basin. Altitudes range from 180 to 1,178 m above sea level, with Sveta Gera (1,178 m above sea level) as the highest peak. **b** Schematic representation of the Bedara cave. From the entrance (1) measuring 0.4 × 0.8 m to the location of the find, the passages are of mostly knee-shaped and pit-like morphology. The current entrance and passage are located at a depth of 16 m (2). The cave features several vertical passages without connection to the surface, such as the one at the discovery site, at a depth of 47 m (4), where also a constant flow of drip water exists (5). Further skeletal remains may have been washed away into the deeper parts of the cave (6)

**Fig. 2:**
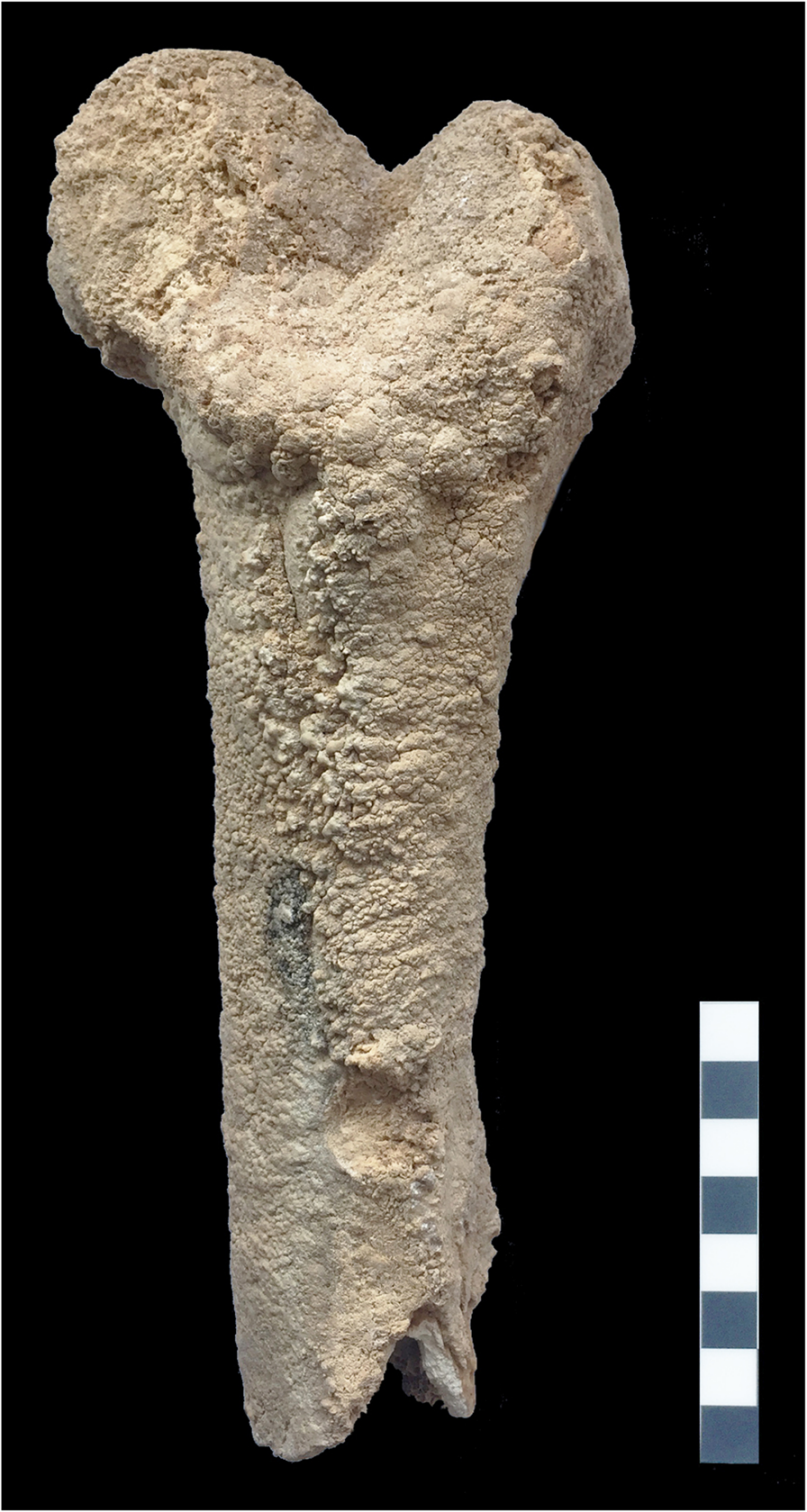
Photograph of the preserved fragment of a proximal femur fragment completely covered by a thick layer of calcium carbonate. The gross anatomical identification and the orientation of the bone are evident due to the specimen’s overall morphological characteristics; a thick concretion covering the entire bone fragment, however, obscures all finer morphological features

The Bedara cave is located on Mt. Žumberačka Gora (NW Croatia) [1] (Fig. 1a). The area lies at the transition between the southeastern Alps, the northwestern Dinarides and the Pannonian Basin. Altitudes range from 180 to 1,178 m above sea level, with Sveta Gera (1,178 m above mean sea level) as the highest peak. Due to its predominantly carbonate-rich composition, this area tends to form karst [2]. Consequently, numerous karst relief forms, both on the surface, and underground are known, including 205 registered speleological features (pits and caves) [3]. The Bedara cave was discovered in 2002 by cavers of the Caving Club Samobor. It is one of the largest caves of northwestern Croatia, with 1,593 m of explored passages reaching down to a depth of 113 m. Sediments occur as speleothems, clay sediments, silt, sand, debris, gravel, and stone blocks. The cave features several vertical passages without a connection to the surface, as seen at the discovery site at a depth of 47 m (Fig. 1b, point 4), where there is also a continuous flow of drip water (from a narrowing in Fig. 1b, point 5). Here, a proximal part of a right femur, heavily encrusted in calcite carbonate, was discovered during a cave research trip in 2017 within a large calcite deposit (Fig. 2). Based on its external morphology, it could have been of human origin. Further animal skeletal remains were found in this location, suggesting that at a certain period, a vertical passage, functioning as a natural trap, had existed.

### The specimen

The specimen, a proximal femoral fragment with a transversely broken shaft, is completely covered by a thick layer of calcium carbonate. Whilst the anatomical identification and the orientation of the bone were relatively evident due to overall morphological characteristics (generic shape consisting of a cylindrical shaft and spherical head), the thick surrounding concretion altered its relative size, giving the impression of a larger animal (or human), and hid all finer morphological characteristics. A precise taxonomic identification through standard anatomical analysis was, therefore, impossible without prior cleaning of the bone surface. To avoid aggressive chemical or mechanical removal of the calcium carbonate, likely damaging the bone fragment, we used clinical computed tomography (CT) to scan the specimen, and then virtually cleaned the obtained volumetric dataset. Thereby, we revealed the bone fragment’s original morphology and partially also its original surface texture.

## Methods

### Radiographic analysis and virtual removal of speleothemic deposits

**Fig. 3:**
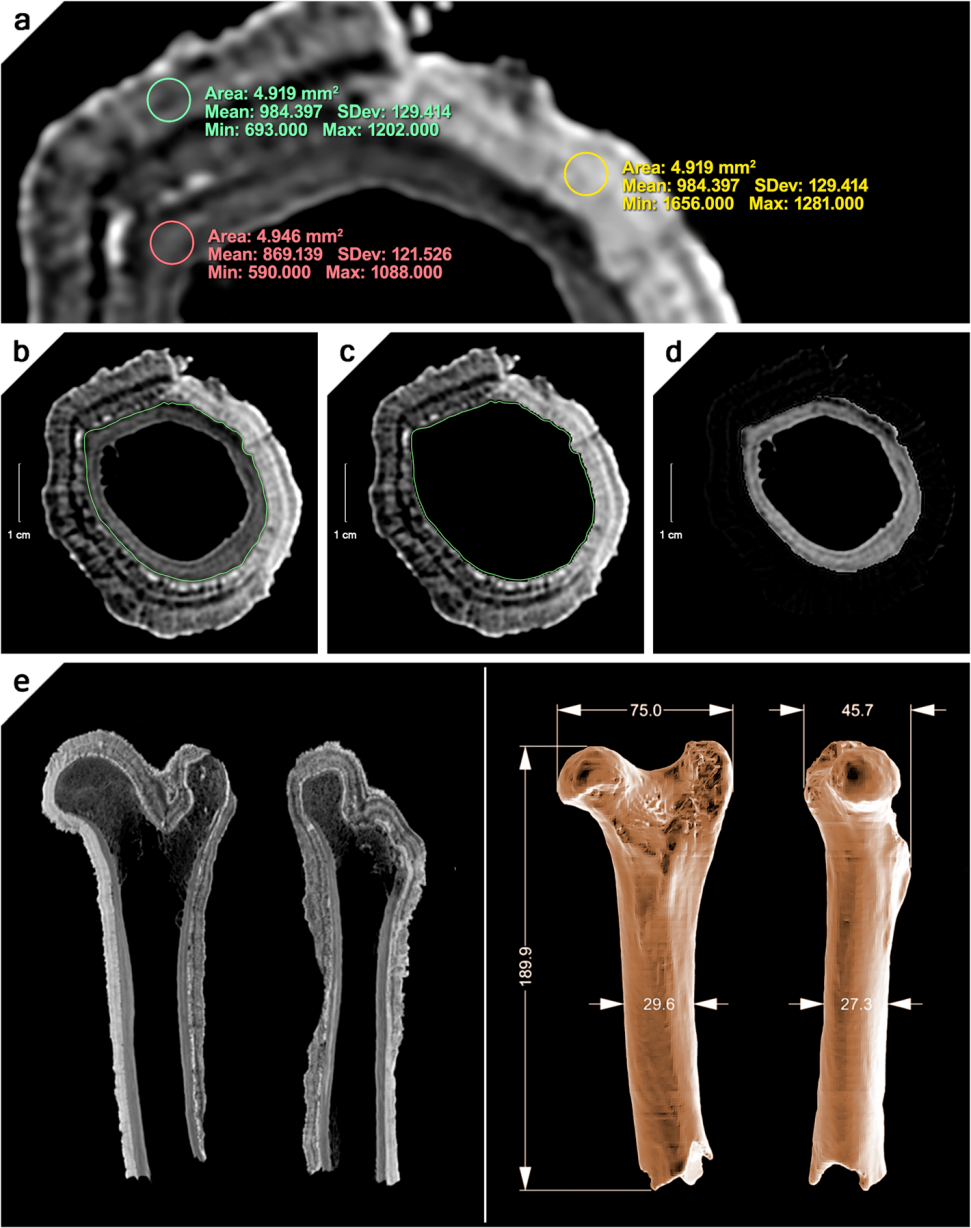
Radiographic analysis and virtual removal of speleothemic deposits. **a** Segmentation of the incrusted bone fragment was a particular challenge due to the sometimes very similar densities of the partially fossilised bone fragment and the surrounding calcite mineral. **b**–**d** A combined manual and HU-threshold based segmentation protocol, previously developed for such applications by one of the authors (P.E.), was therefore applied. **e** Osteological measurements from one of the virtual 3D bone models. The model differing the least from the remaining two models was chosen (Reader 1). Correlative cross-sections from the CT scan are represented. The mean difference between virtual osteological measurements and direct radiological measurements is 0.4 mm ± 0.3 mm (mean ± standard deviation)

The specimen was brought to the University Hospital Centre “Zagreb” (Zagreb, Croatia) and imaged with a clinical computed tomography (CT) scanner (Light Speed Ultra, General Electric, Chicago, USA) using 8 × 1.25 mm collimation, 0.75 pitch, and 120 kVp. Image contrast was considered insufficient for segmentation in the preview on the workstation, and no reconstructions were performed. A different clinical CT scanner (Sensation 40, Siemens Healthineers, Erlangen, Germany) with 40 × 0.6 mm collimation, 0.9 pitch, and 140 kVp at 390 mAs was used for a second scan, producing substantially better contrast (Fig. 3a). Axial sections were reconstructed with a bone weighted kernel (B70s), a slice thickness of 0.6 mm, and an increment of 0.3 mm. A total of 682 slices was generated for coverage of the entire sample.

Nevertheless, segmentation of the incrusted bone remained a particular challenge due to the sometimes very similar densities of the partially fossilised bone fragment and surrounding concretions (Fig. 3a). Hounsfield units (HU) threshold-based or region-growing-based segmentation algorithms were not practicable. A combined, manual and HU threshold-based segmentation protocol, newly developed for this project by one of the authors (PE), was therefore applied. For later validation, segmentation was independently carried out by three readers (R1–3) using the dedicated open-source medical DICOM image viewing software (Horos, v3.2.1, www.horosproject.org): First, in an axially reconstructed series of 702 slices, a free-form region of interest (ROI), most closely corresponding to the demarcation of the bone fragment, was manually drawn on every tenth slice. Particular care was taken not to include components of the calcite crust or omit parts of the bone fragment; furthermore, ROIs were drawn clockwise starting at a twelve o’clock position (Fig. 3b). Second, missing ROIs were interpolated on the remaining slices. After this step, added ROIs were checked by scrolling through the entire data set and manually corrected where necessary. Once each slice contained a ROI circumscribing the bone fragment’s delimitation, the “ROI Volume/Compute Volume...” function allowed a first visual inspection of the virtually cleaned bone fragment. Third, the contents of every ROI on each slice, *i.e.*, the actual bone fragment, was deleted with the “ROI Volume/Erase Content” function, and the resulting data set was saved as a new DICOM series (Fig. 3c). The newly generated and stored template DICOM series, only representing the calcite concrement, was then subtracted from the original (unaltered) axially reconstructed series, resulting in a dataset only showing containing the virtually cleaned femoral fragment (Fig. 3d). Last, using conventional HU threshold segmentation, a three-dimensional (3D) surface rendering was created and exported in the “standard tessellation language” file format for subsequent evaluation and dimensioning with the 3D design software (Rhinoceros 3D, Version 5.4.1, Robert McNeel & Associates, Seattle, WA, USA) (Fig. 3e).

### Taxonomic assessment

The taxonomic assessment was performed by a zooarchaeologist (SR), based on measurements and renderings of the virtual 3D models resulting from CT image segmentation. Measures were taken following von den Driesch [4], and results were then compared with published metric data for species in question. Anatomical terminology used follows standards referenced in the Nomina Anatomica Veterinaria (5th edition) [5].

### ^14^C radiocarbon dating

A direct radiocarbon date of a small sample of the exposed shaft was conducted in conventional 14C age, and fraction corrected using AMS ^13^C. Calibrated age ranges using Intcal13.14c (1 sigma) [6] were 3637–3627 (probability distribution 0.133), 3591–3527 (probability distribution 0.867), (2 sigma) 3647–3516 (probability distribution 0.976), 3408–3405 (probability distribution 0.003) and 3398–3384 (probability distribution 0.020).

### Statistical analysis

**Fig. 4:**
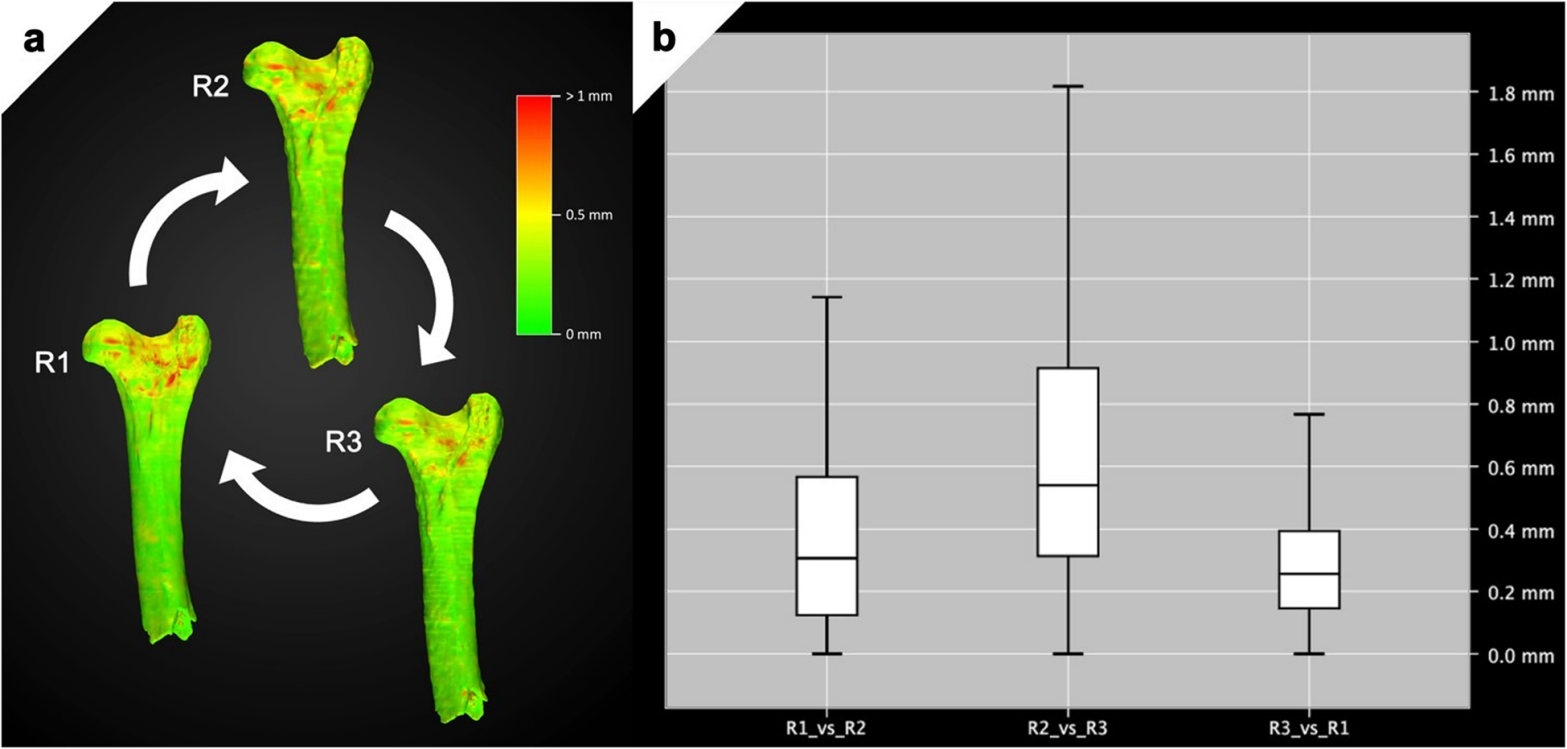
Accuracy of resulting three-dimensional model. **a** Anterior view of resulting three-dimensional bone-models, after the virtual removal of speleothemic deposits, by three independent readers following a combined manual and HU threshold-based segmentation protocol. Projected colour-heat-maps illustrate differences between the respective model and the following model (clockwise). **b** The overall correlation between the three models is excellent, with over 68% of all points lying within a range of less than 1 mm, corresponding to less than three voxels on the computed tomography scan (voxel size = 0.33 × 0.33 × 0.67 mm)

The resulting 3D models of R1–3 were later also statistically compared, by point-to-point comparison of the respective point-clouds, using the dedicated open-source software (CloudCompare V2.6, www.cloudcompare.org) to validate the accuracy of the resulting models (Fig. 4a). For statistical comparison of the inter-reader correlation, the number of points of the point-clouds was reduced to 1 million points per model. Descriptive statistical analysis and boxplots were created using the dedicated software (SPSS Statistics, Version 24, IBM, Armonk, NY, USA), after importing comparative statistical from CloudCompare in the “comma-separated values” data format.

## Results

### Radiographic assessment and virtual cleaning

The combined segmentation protocol proved to be practicable, and the 3D models created by R1–3 achieved excellent congruence, with over 68% of all points lying within a range of less than 1 mm. Mean deviations ranged from 0.29 mm (R3 *versus* R1) to 0.68 mm (R2 *versus* R3). Maximum deviations ranged from 0.8 (R3 *versus* R1) to 2.59 (R2 *versus* R3). Most pronounced deviations between all three models were observed at the epiphyseal level; however, without affecting structures of osteometric relevance (Fig. 4a). Considering that the original CT scan had an anisotropic voxel size of 0.33 × 0.33 × 0.67 mm (*x*, *y*, and *z*, respectively), mean deviations even remain within the limitations of the CT scan’s resolution. In brief, the resulting model provided sufficient data and visualisation for the morphometric and taxonomic study of the specimen (Fig. 3e).

### Osteological description and taxonomic identification

The resulting images show that the bone fragment is well preserved. The only significant alteration can be identified on the greater trochanter, which is damaged, partially crushed, and eroded. The specimen has a massive body, and its shaft is strong and relatively wide. The nutrient foramen is not visible in the scan. The proximal end contains all its features. The femoral head is complete and strongly curved, whilst the *fovea capitis* is small and relatively round. Due to the bulging of the head, the neck is distinct and well defined. The greater trochanter is massive but not too bulky, and damaged, as noted above. Nevertheless, this did not much affect its overall height; evidently, it originally reached the level of the head or slightly extended above it. The trochanteric ridge is curved and closes the trochanteric fossa, which is vertical and deep. The lesser trochanter is faint but visible whilst the third trochanter is absent. The caudal side of the shaft shows a rough surface of the *linea aspera*, extending from the proximal towards the distal end, clearly defined by a lateral ridge and medial ridge. The distal portion of the bone is broken just above the medial and lateral supracondylar tuberosity and missing.

The above-described morphological features are characteristic for the family Suidae (pigs), in particular for the genus *Sus* [7, 8]. The question remains, which of the two species did it belong: domestic (*Sus domesticus*) or wild (*Sus scrofa*)? Both species are anatomically very similar, and often not distinguishable in faunal material [9]. For postcranial elements, morphological criteria are suggestive at best. More reliable metric data require measurable samples, preferably from an adult individual. Both epiphyses at the proximal ends are fused, indicating an adult animal. Although we are limited to a single element, fortunately, measurements of the hindlimbs show less age or sexual dimorphism-related variability than other postcranial elements [10]. Three appropriate measures could be taken (breadth of the proximal end, depth of femoral head, and the smallest diameter of the diaphysis) and compared to wild boar specimens from the reference collection at the Institute for Quaternary Palaeontology and Geology in Zagreb and to metric data for European domestic and wild pigs of the broader region (Radović S, personal data base) [10, 11]. All three parameters (breadth of the proximal end = 75.0 mm, depth of femoral head = 30.7 mm, and smallest diameter of the diaphysis = 29.6 mm) (Fig. 3e) fall within the size range for wild boars.

### ^14^C radiocarbon dating

The direct radiocarbon date of the shaft sample was 4,781 ± 35 years before present, corresponding to the timeframe of the Middle Eneolithic Retz-Gajary culture in the region, based on a series of radiometric dates [12].

## Discussion

Caves can be important windows to the past, in our case the Middle Eneolithic, and for this reason, archaeologists and palaeontologists explore them. Although often better preserved than in open-air sites, skeletal finds in caves are quite frequently encrusted in mineral deposits. The remarkably complete Neanderthal skeletal remains from the Lamalunga cave near Altamura, Bari, in southern Italy [13, 14], dated to between 130,000 and 170,000 years before present are a prominent example [15]. Such circumstances challenge us with the decision of whether to remove the surrounding concrements risking to damage the specimen. It is, therefore, of great importance to find new ways to study such specimens whilst causing minimal or no damage. Radiology has been an advantageous technique in the study of bioarcheological remains, ever since its first use in the study of Egyptian mummies [16], or the Neanderthal remains from Krapina [17, 18]. The excellent resolution of the presented approach can reveal the original shape and surface of an encrusted bone (or even a tooth), allowing anatomical and taxonomic identification, including metric analysis. However, it does not stop there. Even geometric morphometric analyses would be possible from the resulting triangulated 3D models. Nevertheless, the here described procedure shall not replace a traditional approach to osteological/osteometric studies, but, although somewhat demanding and time-consuming, allows the examination of such “hidden” specimens in detail, without the bias otherwise imposed by their actual inaccessibility. The major limitation of our study is that we examined a single and very exceptional specimen, inevitably also limiting comparability with related studies involving computed tomographic analysis of fossilised prehistoric hominin or animal skeletal remains [15, 19–21].

In conclusion, we reported on a novel approach to examine fossil skeletal remains (or even teeth) partially covered or surrounded by mineral deposits, showing that their original shape and surface can be non-invasively revealed in great detail, to allow anatomical and taxonomic identification, including metric analysis. Consequently, by combining clinical computed tomography, a specially developed segmentation protocol, and virtual visualisation and dimensioning, we gained a comprehensive insight into an extraordinary skeletal find from the Middle Eneolithic while leaving it in a “safe environment”, *i.e.*, within its calcite enclosure. This study may, therefore, provide a reference for future palaeontological and anthropological analyses, seeking to unlock the enormous potential of anatomical studies of comparable skeletal remains that are either petrified or enclosed in speleothemic deposits.

## Data Availability

Data is available from the corresponding author upon request.

## Abbreviations

3D: Three dimensional
CT: Computed tomography
DICOM: Digital imaging and communications in medicine
HU: Hounsfield units
R1–3: Readers 1–3
ROI: Region of interest
